# Louisville Medical College

**Published:** 1874-04

**Authors:** 


A CORRESPONDENT writes us from Louisville, Kentucky: "I have just witnessed the closing exercises of the Louisville Medical College for the session of 1873–4. There were seventy-six graduates, and in this number we recognized from the proud old State of Georgia Messrs. J. E. Dupree, E. D. Little and W. B. Mathews. The latter was salutatorian for the occasion, and well may we as Georgians feel proud of him, for in his address he did honor to the Empire State of the South. The college is in a flourishing condition, and has a corps of able men as teachers of medicine. B."

